# Comparison of Incidence of Adjacent Segment Pathology between Anterior Lumbar Interbody Fusion and Transforaminal Lumbar Interbody Fusion Treatments for Lumbosacral Junction

**DOI:** 10.3390/tomography7040072

**Published:** 2021-12-02

**Authors:** Po-Kuan Wu, Meng-Huang Wu, Cheng-Min Shih, Yen-Kuang Lin, Kun-Hui Chen, Chien-Chou Pan, Tsung-Jen Huang, Ching-Yu Lee, Cheng-Hung Lee

**Affiliations:** 1 Department of Education, Taichung Veterans General Hospital, Taichung 40705, Taiwan; b101102049@tmu.edu.tw; 2 Department of Orthopedics, Chi Mei Medical Center, Tainan 71004, Taiwan; 3 Department of Orthopedics, Taipei Medical University Hospital, Taipei 11031, Taiwan; maxwutmu@tmu.edu.tw (M.-H.W.); tjdhuang@tmu.edu.tw (T.-J.H.); lee161022@tmu.edu.tw (C.-Y.L.); 4 Department of Orthopaedics, School of Medicine, College of Medicine, Taipei Medical University, Taipei 11031, Taiwan; 5 Department of Orthopedic Surgery, Taichung Veterans General Hospital, Taichung 40705, Taiwan; stargate@mail.vghtc.gov.tw (C.-M.S.); khc@vghtc.gov.tw (K.-H.C.); pcchou@vghtc.gov.tw (C.-C.P.); 6 College of Medicine, National Chung Hsing University, Taichung 40227, Taiwan; 7 Department of Physical Therapy, Hungkuang University, Taichung 433304, Taiwan; 8 Graduate Institute of Athletics and Coaching Science, National Taiwan Sport University, Taoyuan 333325, Taiwan; robbinlin@ntsu.edu.tw; 9 Department of Nursing, Jenteh Junior College of Medicine, Nursing and Management, Miaoli 45664, Taiwan; 10 Department of Computer Science & Information Engineering, College of Computing and Informatics, Providence University, Taichung 43301, Taiwan; 11 Department of Biomedical Engineering, College of Intelligent Technology, HungKuang University, Taichung 433304, Taiwan; 12 Department of Rehabilitation Science, Jenteh Junior College of Medicine, Nursing and Management, Miaoli 45664, Taiwan; 13 Department of Food Science and Technology, Hungkuang University, Taichung 433304, Taiwan

**Keywords:** adjacent segment pathology, anterior lumbar interbody fusion, transforaminal lumbar interbody fusion, lumbosacral fusion

## Abstract

This research compared the incidence of adjacent segment pathology (ASP) between anterior interbody lumbar fusion (ALIF) treatment and transforaminal lumbar interbody fusion (TLIF) treatment. Seventy patients were included in this retrospective study: 30 patients received ALIF treatment, and 40 patients received TLIF treatment at a single medical center between 2011 and 2020 with a follow-up of at least 12 months. The outcomes were radiographic adjacent segment pathology (RASP) and clinical adjacent segment pathology (CASP). The mean follow-up period was 42.10 ± 22.61 months in the ALIF group and 56.20 ± 29.91 months in the TLIF group. Following single-level lumbosacral fusion, ALIF is superior to TLIF in maintaining lumbar lordosis, whereas the risk of adjacent instability in the ALIF group is significantly higher. Regarding ASP, the incidence of overall RASP and CASP did not differ significantly between ALIF and TLIF groups.

## 1. Introduction

Adjacent segment pathology (ASP) is a major adverse event of lumbar or lumbosacral fusion. According to a biomechanical study, the lumbosacral junction is the most critical segment for sagittal alignment [1]. After lumbar fusion, the lumbar segments adjacent to the fused vertebra will inevitably bear more pressure [2] and require a more substantial range of motion [3] to maintain the patients’ daily activity. Consequently, ASP may occur, which includes disc degeneration, listhesis, instability, and compression fracture. ASP can be further divided into radiographic ASP (RASP), which refers to adjacent segment radiographic change, and clinical ASP (CASP), which refers to symptoms at adjacent segments associated with RASP [4]. According to a relevant systematic review [5], the incidence of RASP after lumbar fusion ranges from 10.8% to 100% (3–19.5% per year), and that of CASP ranges from 2.6% to 30.3% (0.4–5.1% per year). The risk factors for ASP can be divided into pre-existing factors (age, sex, osteoporosis diagnosis, smoking status, physical activity level, obesity status, menopause diagnosis, bone mineral density, and pre-operation pathology at adjacent segment) and surgery-related factors (postoperative sagittal alignment, adjacent segment operation, the position of pedicle screws, and floating fusion) [6,7,8].

ASP can considerably lower patients’ quality of life and increase the need for revision surgery [5,9]. With the dramatically growing number of lumbar fusions performed in recent years [10], ASP is becoming more widespread. A deeper understanding of this late complication is therefore essential. Studies focusing on the risk factors for ASP have been conducted to expand knowledge on the prevention of ASP. Limiting the damage to the adjacent segment, preserving the posterior column integrity, and restoring the lumbar lordosis are protective factors against the development of ASP for single-level lumbar fusion [11,12,13,14].

Studies analyzing the relationship between various surgical methods and ASP have not reached a clear consensus. Anterior interbody lumbar fusion (ALIF) allows access to the entire disk, leading to a larger fusion cage and a lower subsidence rate [15]. Transforaminal lumbar interbody fusion (TLIF) has lower vascular risk, less soft tissue damage, and more uncomplicated surgical techniques [16]. ALIF and TLIF are associated with more successful ASP prevention than posterior lumbar interbody fusion (PLIF) treatment due to reducing damage to the integrity of the posterior complex [13,17]. Compared to TLIF, ALIF is superior in restoring lumbar and segmental lordosis, which are the protective factors of ASP [18,19]. However, no studies have compared the incidence of ASP between ALIF and TLIF treatments at the lumbosacral junction. This study intends to address the research gap.

## 2. Materials and Methods

### 2.1. Patient Population

**Figure 1:**
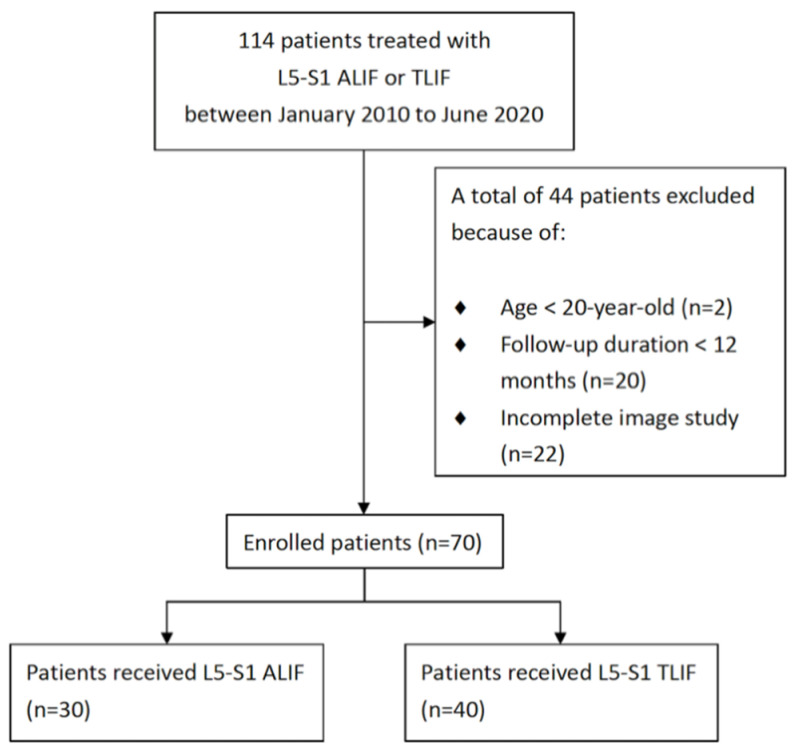
Patient inclusion flow chart.

This retrospective study reviewed the medical records and radiographic images of 70 patients who received interbody lumbar fusion. Refractory and disabling back pain with degenerative discs, spondylolisthesis, spinal stenosis, or scoliosis are indications of spinal fusion [20]. The interbody fusion technique was decided by the patients after the surgeon explained the advantages and disadvantages of ALIF and TLIF treatments. The diagnosis of the patients in this study included spondylolisthesis, herniated intervertebral disc, and spinal stenosis at the L5-S1 level. All patients underwent the operation at a single medical center between 2011 and 2020, and Figure 1 displays the inclusion flow chart. The inclusion criteria were as follows: (1) being aged ˃20 years, (2) receiving single-level ALIF or TLIF treatment at L5–S1, and (3) having a follow-up duration ˃ 12 months. The exclusion criteria were as follows: (1) receiving revision surgery after lumbar fusion, (2) receiving lumbar operation at any level of the lumbar spine other than L5–S1, (3) having a diagnosis of nonunion after lumbar fusion at L5–S1, and (4) having a spinal deformity related to malignancy, trauma, or infection. This study was approved by the Institutional Review Board of Taichung Veterans General Hospital (No CE21228A).

### 2.2. Surgical Procedures

In ALIF treatment, patients were placed in a supine position. After creating a midline lower abdominal incision, the surgeon used a retroperitoneal approach to expose the L5–S1 space. The prevertebral fascia and anterior longitudinal ligament with thickened fibrotic anterior annulus were removed. After endplate preparation, the cage packed with allograft was impacted into the interbody space under fluoroscopy. We closed the wound and subsequently turned the patient to a prone position. The Wiltse muscle-sparing approach was adopted for soft tissue dissection, and pedicle screws with lordotic rods were placed at the L5–S1 level to create more lordosis and stability. Laminotomy was performed at the L5–S1 level in some instances with severe symptoms. The wound of the posterior approach was closed after the instrumentation, and spinal alignment was confirmed with radiography.

In TLIF treatment, patients were placed in a prone position. After a midline skin incision over the lower back was made, soft tissue was dissected through the paramedian muscle until the posterior elements of the vertebral body were exposed. The surgeon then performed partial laminectomy and facetectomy. The spinal disc at L5–S1 was removed, and the cage packed with allograft was subsequently inserted. Then, we applied pedicle screws with the same method as that used in ALIF treatment.

### 2.3. Radiological Assessment and Clinical Record

**Figure 2:**
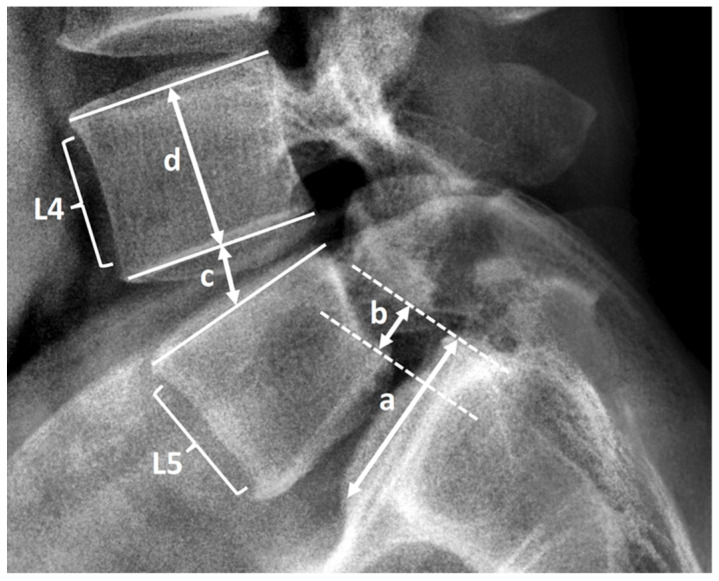
Lumbar lateral radiograph exhibits examples of L5–S1 vertebral body slippage, disc height, and vertebral body height measurement. Line segment a indicates the superior endplate of S1. Line segment b indicates vertebral body slippage length at L5–S1. Line segment c marked the distance between the midpoint of the inferior endplate of L4 and the superior endplate of L5, which indicates L4–5 disc height. Line segment d marked the distance between the midpoint of the superior endplate of L4 and the inferior endplate of L4, which indicates L4 vertebral body height.

**Figure 3:**
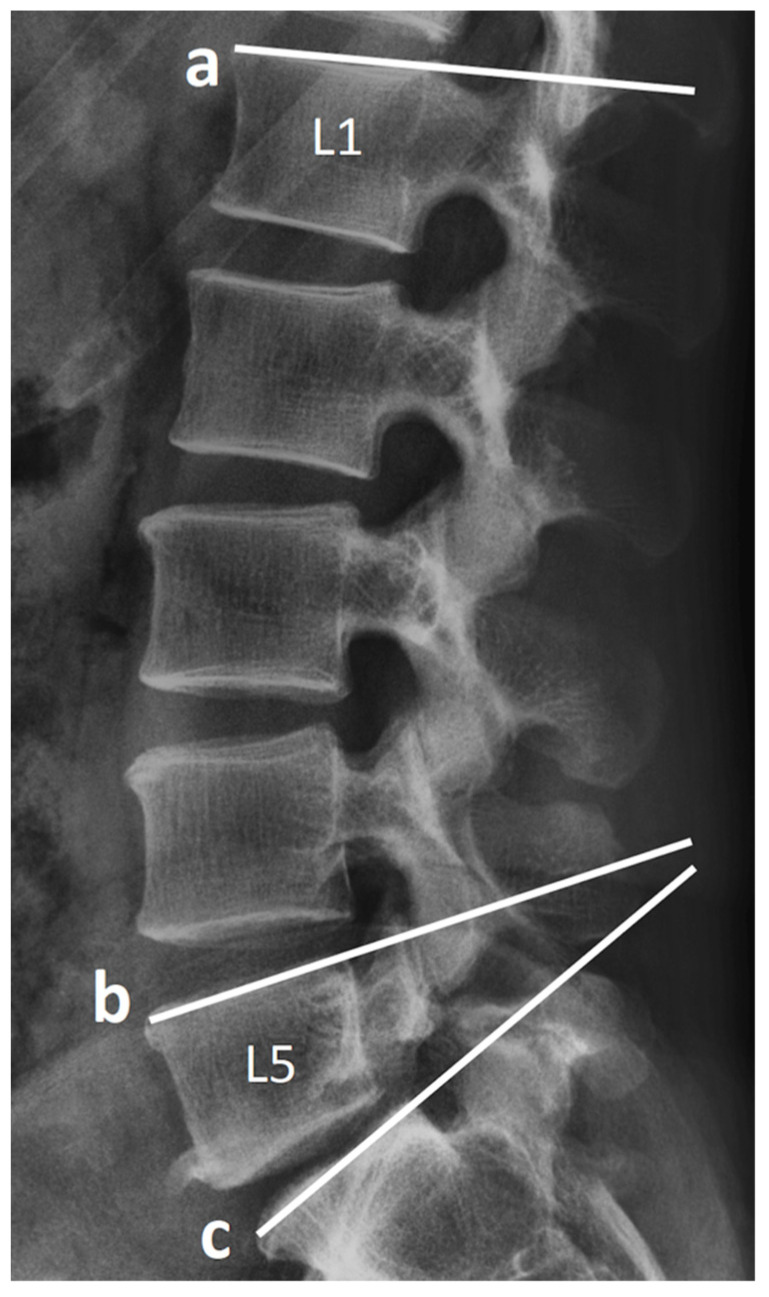
Lines a, b, and c parallel the superior endplates of L1, L5, and S1, respectively. The included angle between line a and line c indicates the measurement of lumbar lordosis. The included angle between line b and line c indicates the measurement of segmental lordosis.

Clinical and demographic data of age, sex, body weight, body height, body mass index, smoking status, bone marrow density (T score), American Society of Anesthesiologists grade, blood loss, operative time, and follow-up duration were collected from medical records. After the operation, the patients were followed up with radiographic evaluation every 3 months until the fusion of the spine was confirmed, and then every 12 months afterward. The patients’ lumbar spinal radiographs with anteroposterior, lateral, flexion, and extension view were evaluated using Surgimap software (Nemaris, New York, NY, USA). We assessed the following preoperative and postoperative radiographic parameters of the lumbar spine: pelvic tilt (PT), lumbar lordosis (LL), L5–S1 segment lordosis (SL), lumbopelvic mismatch (pelvic incidence minus LL), disc height, and vertebral body slippage length over L5–S1. The method of measurement is presented in Figure 2 and Figure 3. Two authors are responsible for radiographic measurement.

Adjacent segments in this study were defined as two cephalad lumbar motion segments above the L5–S1 spinal fusion [21]. Radiographic ASP was defined as image evidence of one or more of the following spinal lesions, which did not occur before L5–S1 lumbar fusion at L3–L4 or L4–L5 level: (1) disk degeneration (average disk height reduction ˃ 10%), (2) listhesis (anterior or posterior vertebral body slippage ˃ 4 mm), (3) compression fracture (anterior or posterior vertebral body height reduction ˃ 20% compared with unaffected portion), and (4) instability (angular motion ˃ 10°) [7,12,17,22]. The data of length measurement on the radiographs were calibrated with the actual length of implants by using the integrated function of the Surgimap software to make the radiographs more compatible at diverse time points. CASP was defined as newly developed symptoms accompanied by radiographic change after postoperative symptom relief for at least six months [4]. Hospitalization, outpatient center, and telephone interview records were collected and compared with the radiography for CASP evaluation.

### 2.4. Statistical Analyses

The two groups were compared using an independent t-test for continuous variables and a chi-square test for categorical variables. We used the log-rank test for the survival analysis of CASP. A *p* value of <0.05 was considered significant for the analyses. Statistical analyses were performed using SAS (version 9.4M7, SAS Institute, Cary, NC, USA).

## 3. Results

### 3.1. Demographics and Characteristics of Patients

From 2011 to 2020, 70 patients received L5–S1 single-level lumbar fusion at our institution; 30 (42.8%) and 40 (57.2%) patients received ALIF and TLIF treatment, respectively. Table 1 lists the demographics and characteristics of both groups.

Preoperatively, PT, PI-LL, LL, and SL were not significantly different between the two groups. Postoperatively, LL was significantly larger in the ALIF group (ALIF, 45.47° ± 13.78°; TLIF, 33.17° ± 12.52°; *p* < 0.001). The intraclass correlation coefficient (ICC) of radiographic measurements was evaluated using the guideline outlined by Koo et al. [23] For postoperative LL, the ICC was 0.94, which revealed excellent agreement between the two measurements.

In terms of surgical outcomes, operation time, blood loss, and complications were analyzed. The anesthesia duration of ALIF treatment (including switching the position from supine to prone) was 372.33 ± 87.73 min, and that of TLIF treatment was 233.38 ± 60.59 min (*p* < 0.001). Blood loss was 410 ± 523.32 cc in the ALIF group and 420.77 ± 325.51 cc in the TLIF group (*p* = 0.92). One patient in the TLIF group experienced L5–S1 surgical-site hematoma with symptoms of nerve root compression after the operation, and the signs were relieved after wound debridement.

### 3.2. RASP

**Figure 4:**
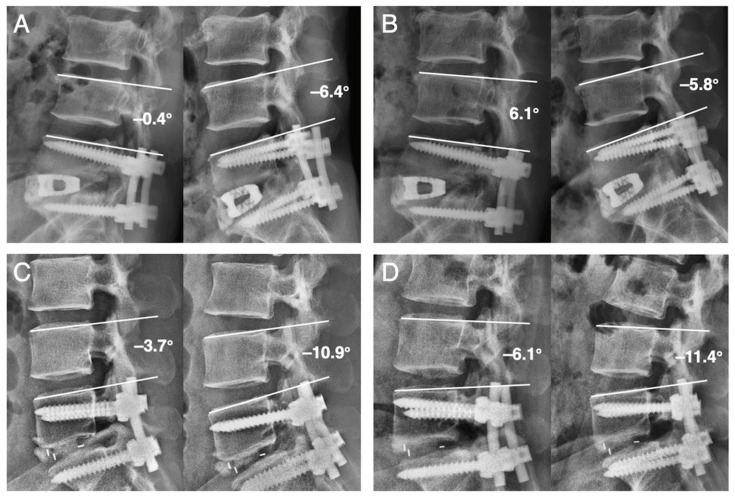
Dynamic lateral radiographs exhibit examples of development/non-development of instability (angular motion > 10°) at L4-L5 in patients who received ALIF or TLIF treatment. Images were obtained from a 48-year-old man who received L5–S1 ALIF (**A**,**B**) and a 41-year-old woman who received L5–S1 TLIF (**C**,**D**). (**A**) Postoperative radiographs exhibiting normal angular motion at L4–L5 (6°). (**B**) 12-month follow-up radiographs showing 11.9° angular motion at L4–L5 with definitive evidence of instability. (**C**) Postoperative radiographs showing normal angular motion at L4–L5 (7.2°). (**D**) 12-month follow-up radiographs exhibiting 5.3° angular motion at L4–L5 without definitive evidence of instability.

A comparison of the incidence of RASP is presented in Table 2. At the L4–L5 level, the incidence of RASP was 63.3% in the ALIF group and 50% in the TLIF group (*p* = 0.27). However, instability was observed more frequently at the L4–L5 level in the ALIF group (ALIF 43.3%; TLIF 15%; *p* = 0.008). Figure 4 displays examples of development and nondevelopment of instability at the L4–L5 level. Other classifications of RASP, namely disc degeneration, L4 compression fracture, and listhesis, did not differ significantly between the two groups. At the L3–L4 level, the incidence of RASP was 53.3% in the ALIF group and 47.5% in the TLIF group (*p* = 0.63). Other classifications of RASP, namely disc degeneration, L4 compression fracture, listhesis, and instability, did not differ significantly between the two groups. The mean onset time of RASP is 37.92 ± 22.41 months in the ALIF group and 40.63 ± 25.69 months in the TLIF group (*p* = 0.69).

Detailed radiographic data of RASP at the final follow-up was also analyzed (Table 3). The angular motion over L3–L4 in the ALIF group was 9.31° ± 3.31°, and that in the TLIF group was 7.52° ± 3.79°, with significant difference (*p* = 0.04). The angular motion over L4–L5 in the ALIF group was also larger than that in the TLIF group, but no significant difference was observed (ALIF 12.03° ± 4.25°; TLIF 10.09° ± 4.89°; *p* = 0.09).

### 3.3. CASP

**Figure 5:**
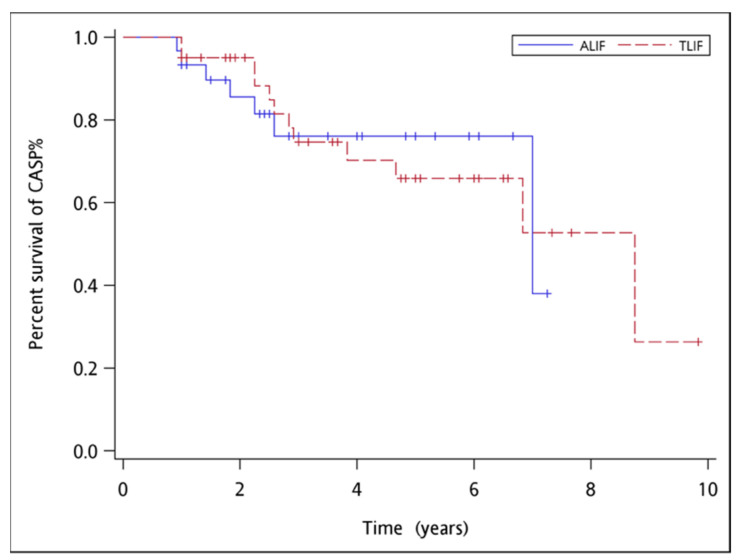
Kaplan–Meier survival curve of ALIF versus TLIF treatment.

Survival analysis of CASP incidence revealed no significant difference between the two groups (Table 4). The 5-year disease-free survival rate was 68% in the ALIF group and 35% in the TLIF group (*p* = 0.57). Figure 5 is the Kaplan–Meier survival curve of ALIF versus TLIF treatment. Only one patient in the ALIF group received oblique lumbar interbody fusion as revision surgery at L3-5 level due to the development of CASP seven years after the previous lumbosacral fusion.

## 4. Discussion

ASP occasionally becomes symptomatic and is the principal cause of revision surgery after lumbar fusion [24]. Studies focusing on the risk factors for ASP have been conducted to expand knowledge on the prevention of ASP. Both ALIF and TLIF treatments seem to have protective characteristics against ASP [13,15,16,17]. This study aimed to compare the incidence of ASP between ALIF and TLIF treatment for the lumbosacral junction.

### 4.1. Lumbar Lordosis

Lau et al. conducted a meta-analysis in 2021 and concluded that the decreased preoperative and postoperative lumbar lordosis are risk factors for ASP [19]. The lumbosacral junction is the most critical segment for sagittal alignment [1]. Most LL occurs from L4 to S1, and restoring lower LL could prevent ASP [11]. Dorward et al. indicated that ALIF treatment is superior to TLIF treatment for correcting SL [25]. Nevertheless, single-level lumbar fusion to correct overall LL is challenging [16]. In our study, ALIF treatment was superior to TLIF treatment in maintaining LL with a mean gain in lordosis of 2.28° compared with a 6.34° loss for the TLIF group after lumbosacral fusion. The postoperative LL is significantly larger in the ALIF group, but the overall incidence of ASP between groups showed no significant difference.

### 4.2. Instability

After patients received ALIF or TLIF treatment at L5–S1, the RASP was evaluated at L4–L5 and L3–L4 levels. We found that the overall incidence of RASP did not differ significantly between the two groups. However, at the L4–L5 level, the instability rate, defined as angular motion >10°, was significantly higher in the ALIF group. Although the instability rate at L3–L4 between groups did not differ significantly, the ALIF group’s L3–L4 angular motion was significantly larger than that of the TLIF group at the final follow-up. A biomechanical study of lumbar fusion reported that the facet contact patterns at the adjacent segment changed, and the segmental motion increased to compensate for the decreased range of motion at the instrumented level [3]. A more rigid instrumented lumbar fusion will result in a higher risk of adjacent instability [26,27]. ALIF devices generally had higher strength, stiffness than TLIF [28]; therefore, this difference may contribute to the higher instability rate at the adjacent segments in the ALIF group.

### 4.3. CASP

A systematic review of the clinical outcome of ALIF and TLIF treatments in 2018 revealed that the visual analog scale score for back pain did not differ significantly between the two groups [29]. In our study, the incidence of CASP, which was defined as compatible clinical symptoms and radiographic change, did not differ significantly between the groups, and this lack of difference is congruent with the finding of the research mentioned above. Park et al. found the incidence of facet violation after pedicle screw placement was around 50% [30]. Furthermore, injury to the adjacent level structures during instrumenting pedicle screws is related to the development of ASP [31]. As reported by Pourtaheri et al. [32], pedicle screw placement may cause substantial paraspinal muscle disruption and eventually muscle atrophy related to back pain. Ajiboye et al. indicated that ALIF treatment without pedicle screws might have a superior clinical outcome and less pain than TLIF treatment [29]. All of the patients included in our study underwent pedicle instrumentation to achieve circumferential spinal stability, which may be one of the reasons for the similar incidence of CASP between the groups.

### 4.4. Limitations

Our study has particular limitations. First, we employed a retrospective design, and the sample size was relatively small. Second, although the baseline characteristic did not differ significantly between the two groups, the following period significantly differed. However, this point will not affect the study result. A future prospective study should be done to answer this question. Third, our study design did not include patient-reported outcome measures such as the Oswestry disability index, so we cannot provide such quantified clinical outcomes in this retrospective study. Fourth, we did not arrange regular magnetic resonance imaging or computed tomography examinations for the patients during follow-up; consequently, some types of RASP, namely facet joint degeneration, herniated disc, and spinal stenosis, could not be evaluated. Because of this lack of comprehensive evaluation, the incidence of RASP may be underestimated.

## 5. Conclusions

Following single-level lumbosacral fusion, ALIF is superior to TLIF in maintaining lumbar lordosis, whereas the risk of adjacent instability in the ALIF group is significantly higher. Regarding ASP, the incidence of overall RASP and CASP did not differ significantly between ALIF and TLIF groups.

## Figures and Tables

**Table 1:** Demographic and Clinical Characteristics of Patients.

	ALIF	TLIF	*p* Value
Age (years)	54.60 ± 15.20	54.25 ± 15.34	0.92
Sex (male) *	10 (33.33%)	18 (45.00%)	0.32
Body weight (kg)	63.83 ± 10.86	68.5 ± 12.26	0.10
Body height (cm)	159.5 ± 8.85	161.43 ± 8.95	0.37
BMI	25.02 ± 3.08	26.25 ± 3.97	0.16
Smoking status *	2 (6.67%)	7 (17.50%)	0.18
BMD (T-score)	−1.37 ± 1.16	−1.28 ± 1.39	0.93
Follow-up (month)	42.10 ± 22.61	56.20 ± 29.91	0.03
ASA Grade 1 *	7 (23.33%)	7 (17.50%)	0.90
ASA Grade 2 *	22 (73.33%)	31 (77.50%)	
ASA Grade 3 *	1 (3.33%)	2 (5.00%)	
L5-S1 Spondylolisthesis * ^†^	26	29	0.37
L5-S1 HIVD *^†^	3	7	
L5-S1 Spinal stenosis *^†^	1	4	
Preop PT (°)	21.23 ± 7.48	20.41 ± 8.91	0.80
Preop PI-LL	10.85 ± 8.31	14.46 ± 12.52	0.39
Preop LL (°)	44.05 ± 14.51	38.91 ± 15.93	0.19
Preop SL (°)	16.98 ± 8.02	17.84 ± 7.17	0.65
Preop L5–S1 disc ht (mm)	6.99 ± 2.47	8.13 ± 1.85	0.11
Preop L5–S1 slip (mm)	5.75 ± 4.67	2.31 ± 4.40	0.03
Postop PT (°)	18.28 (5.22)	18.94 (8.29)	0.69
Postop PI-LL	9.56 (6.41)	12.38 (9.92)	0.17
Postop LL (°)	45.47 ± 13.78	33.17 ± 12.52	<0.001
Postop SL (°)	20.20 ± 13.54	16.71 ± 6.35	0.16
Postop L5–S1 disc ht (mm)	12.49 ± 1.89	9.96 ± 1.89	<0.001
Postop L5–S1 slip (mm)	2.15 ± 3.45	1.02 ± 3.14	0.16

Values are mean ± SD or numbers (%) *. ^†^ Preoperative diagnosis. ALIF, anterior lumbar interbody fusion; TLIF, transforaminal lumbar interbody fusion; BMI, body mass index; BMD, bone mineral density; ASA, American Society of Anesthesiologists Classification; preop, preoperative; PI, pelvic incidence; SS, sacral slope; PT, pelvic tilt; PI−LL, pelvic incidence minus lumbar lordosis; LL, lumbar lordosis; SL, segmental lordosis; ht, height; slip, vertebral body slippage length; postop, postoperative.

**Table 2:** Summary of Radiographic Adjacent Segmental Pathology.

	ALIF (*n* = 30)	TLIF (*n* = 40)	*p* Value
RASP at L3–4	16	19	0.63
RASP at L4–5	19	20	0.27
Classification of RASP at L3–4			
Disc degeneration	13	12	0.25
L3 compression fx	1	0	0.43
Listhesis	0	3	0.25
Instability	5	5	0.73
Classification of RASP at L4–5			
Disc degeneration	11	15	0.94
L4 compression fx	1	1	1.00
Listhesis	1	4	0.38
Instability	13	6	0.008

Values are numbers. ALIF, anterior lumbar interbody fusion; TLIF, transforaminal lumbar interbody fusion; RASP, radiographic adjacent segmental pathology; fx, fracture.

**Table 3:** Comparison of Radiographic Detail of Adjacent Segmental Pathology.

	ALIF	TLIF	*p* Value
Level of L3–4			
Last FU − postop/postop disc ht (%)	−10 ± 8.2	−8.2 ± 9.5	0.42
Last FU slip (mm) *	−1.75 ± 1.16	−1.81 ± 1.56	0.85
Last FU angular m. (°)	9.31 ± 3.31	7.52 ± 3.79	0.04
Last FU − postop/postop L3 vertebral body ht (%)	−1.6 ± 3.3	−0.8 ± 1.7	0.24
Level of L4–5			
Last FU − postop/postop disk ht (%)	−8.8 ± 9.5	−7.6 ± 9	0.57
Last FU slip (mm) *	−1.5 ± 1.37	−1.58 ± 1.93	0.85
Last FU angular m. (°)	12.03 ± 4.25	10.09 ± 4.89	0.09
Last FU − postop/postop L4 vertebral body ht (%)	−1.5 ± 3.2	−1.3 ± 3.8	0.78

Values are mean ± SD. * Negative values represent retrolisthesis. ALIF, anterior lumbar interbody fusion; TLIF, transforaminal lumbar interbody fusion; last FU, last follow-up; postop, postoperative; ht, height; slip, vertebral body slippage length; angular m., angular motion.

**Table 4:** Survival Analysis of Clinical Adjacent Segmental Pathology.

	ALIF	TLIF	*p* Value
1-year survival rate	0.92	0.93	0.93
3-year survival rate	0.76	0.81	0.75
5-year survival rate	0.68	0.35	0.57

ALIF, anterior lumbar interbody fusion; TLIF, transforaminal lumbar interbody fusion.

## Data Availability

The data presented in this study are available on request from the corresponding author. The data are not publicly available due to policy of Taichung Veterans General Hospital.
